# The effect and durability of postural education and corrective games on the alignment of the thoracic and cervical spine and the daily habits in children

**DOI:** 10.1038/s41598-025-29071-6

**Published:** 2025-11-30

**Authors:** Majid Barzegari, Ali Shamsi Majelan

**Affiliations:** Corrective Exercises and Sports Injury Department, Faculty of Physical Education & Sport Sciences, University of Guilan, kilometers 10 Rasht-Ghazvin Road, Rasht, 4199613776 Iran

**Keywords:** Posture education, Corrective games, Spine, Posture, Daily activities, Health care, Health occupations, Medical research

## Abstract

This study aims to compare the effects of posture education and corrective games on the alignment of the thoracic and cervical spine, as well as the daily habits in children. This is a three-armed individual-randomized trial design of three groups in blinded evaluators. The statistical population of this study was formed by elementary students with malalignments in the thoracic and cervical spine of Baharestan city (Iran). A total of 60 participants were assigned to this study and using a simple random method with computer-generated random numbers divided into posture education group (PE, *n* = 20) corrective games group (CG, *n* = 20) and control group (CON, *n* = 20) groups. Kyphosis angle, forward head posture and forward shoulder posture measured with a flexible ruler, goniometer, and double square, respectively. Also, daily habits measured with students’ daily functional activities questioner. A repeated measures ANOVA analysis of variance (3 × 3, Group×Time) was utilized to analyze data. Significance was set at *p* ≤ 0.05. Both the PE and CG showed significant improvements in kyphosis (*p* = 0.01 for PE, *p* = 0.02 for CG), forward head posture (*p* = 0.02 for PE, *p* = 0.04 for CG), forward shoulder posture (*p* = 0.001 for PE, *p* = 0.02 for CG), and daily habits (*p* = 0.02 for PE, *p* = 0.03 for CG) after an 8-week training intervention compared to the CON group. Also, after the training period, the analysis revealed no statistically significant differences in the dependent variables between the PE group and the CG, with a p-value greater than 0.05. However, after a 3-month detraining period, the changes in both the PE and CG were found to be statistically insignificant (*p* > 0.05). The interventions effectively enhanced participants’ posture and daily activity patterns, with no significant differences between the PE and CG groups. The sustainability of these improvements indicates that participants developed lasting skills and habits that promote spinal health. This study highlights the importance of integrating educational and engaging physical activities into curricula to support children’s musculoskeletal well-being.

**Trial registration**: IRCT registration number: IRCT20250316065103N1, Registration date: 2025-03-25 (Retrospectively registered), Trial Id: 82539.

## Introduction

Good posture serves as a fundamental indicator of both physical and mental well-being, reflecting an optimal equilibrium among the skeletal, muscular, and joint systems^1^. Proper posture not only enhances physical health but also positively impacts mental well-being by reducing the risk of mood disorders and boosting confidence, thereby fostering a more positive self-image^2^. Poor posture in children can result in significant and long-lasting complications^3^. Studies indicate that inappropriate posture, particularly during growth phases, can adversely affect both the structure and function of the spine^4,5^. For instance, poor sitting habits, excessive forward bending, and improper handling of heavy school bags can lead to abnormalities in various spinal regions, especially the thoracic and cervical areas^6^. A prevalent consequence of poor posture in children is chronic musculoskeletal pain^7^, which can hinder physical activity and negatively impact emotional well-being, ultimately affecting their overall development and quality of life.

Studies indicate that prolonged and improper sitting postures, along with the misuse of computers and smartphones, as well as inadequate techniques for carrying school bags, can lead to chronic pain in the neck, shoulders, and back^8,9^. Consequently, it is crucial to focus on training and correcting physical conditions, particularly during daily activities. Positive or negative alterations in this domain have a direct impact on individuals’ physical and mental states, significantly contributing to the prevention of anomalies and the enhancement of overall quality of life^10^. To address these issues, various interventions have been developed to promote better posture and alleviate associated pain.

Preventing physical disabilities in children through school-based interventions is a crucial and effective strategy for enhancing their health and physical performance^11^. Various interventions have been proposed to improve posture and correct postural abnormalities, with the most significant being posture education and corrective exercises^10^. Posture education serves as a direct and targeted approach to increase awareness of proper physical alignment, rectify postural issues, and reinforce healthy movement habits^12^. By raising awareness and educating children on appropriate behaviors and methods for utilizing their bodies and environments through suitable sports and physical education programs children can learn essential skills, from sitting correctly to properly carrying bags and using technological devices^13^. Research indicates that these programs can lead to substantial improvements in children’s physical posture^14,15^.

Additionally, corrective games offer an innovative approach to posture correction and have gained attention from health professionals for their interactive and engaging nature^16,17^. By integrating physical exercises into a fun format, these games motivate children to increase their physical activity, which contributes to improved posture. Research indicates that such programs can lead to significant enhancements in children’s physical posture^17,18^.

Previous research has demonstrated that corrective games, due to their engaging and interactive nature, can motivate children to be more physically active, thereby aiding in posture improvement^18^. Conversely, posture education focuses directly on enhancing individuals’ awareness of correct posture and correcting abnormalities through the teaching of proper techniques^14^. While both methods contribute to better posture in students, several ambiguities and questions remain unanswered, such as: Which intervention is more optimal and suitable for improving students’ posture and preventing postural abnormalities? Is there a difference in the degree to which these interventions impact students’ posture? Which training program is more sustainable? To address these questions, this study aims to compare the effects and durability of posture education and corrective games on the alignment of the thoracic and cervical spine, as well as the daily habits in children.

## Methods

### Participants

The statistical population of this study comprised elementary students with malalignments in the thoracic and cervical spine from Baharestan city, Iran. The participants were selected based on available samples identified through local schools within the province, ensuring a representative recruitment process. Participants were required to complete all the training sessions and attend all assessment sessions, and were asked to avoid intense physical activities during the study. As a result of these requirements, a total of 60 participants were assigned to this study and using simple and random method with computer-generated random numbers divided into posture education group (PE, *n* = 20) corrective games group (CG, *n* = 20) and control group (CON, *n* = 20) groups. (Table 1). A power analysis was conducted to determine the sufficient sample size of each group. The sample size was calculated based on a previous study by Rajabi, Minoonejad (19) with an alpha level of 0.05, effect size of 0.3 and an actual power (1-beta) of 0.80. The analysis (G × Power, Version 3.1.9.2, University of Kiel, Germany) revealed that a sample size of *n* = 18 would be sufficient for each group to find significant effects between variables. To account for potential participant dropouts, the number of participants was increased by 10% for each group, ensuring adequate power for the study.

The inclusion criteria for the study included: (1) Gender of the participants: male only; (2) Willingness to participants: must express a willingness to take part in the research; (3) Age of participants: should be elementary students (ages 9 to 12); (4) General health: must not have experienced any acute or chronic diseases in the past six months; (5) Parental or legal guardian consent: written consent from a parent or legal guardian is required for participation; (6) No prior participation in similar programs: Participants must not have engaged in similar training programs or other posture-related interventions in the past six months; (7) Spinal status: must be evaluated and approved by a specialist, confirming no significant spinal or neck abnormalities; (8) Ability to attend training sessions: must be capable of attending all training sessions and corrective games; (9) No use of effective medications: should not be taking medications that affect the musculoskeletal system; (10) Absence of psychological issues: must not have psychological problems that could impact their ability to participate in the research. The exclusion criteria for the study were as follows: (1) Regular absence: who miss more than three training sessions or games; (2) Change in health status: who develop acute or chronic diseases during the study period; (3) Failure to comply with instructions: who consistently disobey training guidelines and corrective game protocols; (4) School change: who change schools during the course of the research; (5) Voluntary withdrawal: who choose to discontinue their participation for any reason, with agreement from their parents or guardians. Before the initiation of the study, the participants (children individuals) and their parents were informed about the research procedures, and their written consent was obtained.

**Table 1:** Baseline measurements (mean ± SD).

Characteristic		PE training (*n* = 20)	CG training (*n* = 20)	Control group (*n* = 20)	*p*-value
		Mean ± SD	Mean ± SD	Mean ± SD	
Age	(years)	11.12 ± 1.76	11.76 ± 1.84	11.49 ± 1.39	0.23
Height	(cm)	136.21 ± 7.9	135.2 ± 7.89	136.18 ± 6.99	0.11
Body mass	(kg)	35.43 ± 4.36	36.23 ± 4.32	35.48 ± 5.12	0.23
BMI	(kg/m^2^)	23.19 ± 2.1	23.46 ± 2.76	23.02 ± 2.86	0.46

*Significance level was considered *p* ≥ 0.05.

BMI: Body mass index; PE: Posture education; CG: Corrective games.

### Study design

This study employed a three-armed individual-randomized controlled trial design with convenience sampling. The study followed the CONSORT 2025 guidelines for reporting randomized trials^19^. The participants were pair-matched based on their malalignments and then randomly divided into three groups: posture education group (PE, *n* = 20) corrective games group (CG, *n* = 20) and control group (CON, *n* = 20) groups. The randomization was performed using a computer-generated block randomization table. Randomization was carried out by an independent investigator who was not acquainted with the testing protocol, utilizing a random number table to ensure unbiased group assignment. To further reduce the risk of bias, group allocation was concealed within opaque envelopes until after participants were enrolled in the study. For the purposes of blinding, one researcher administered both the pre-test and post-test assessments, while a different researcher implemented the exercise programs, thereby maintaining the integrity of the study’s design. The experimental groups participated in their programs, which involved 60-minute sessions, 2 days per week, for a duration of 2 months. The control group performed their regular daily activity. The study period spanned 11 weeks, with the following timeline: Week 1 - Familiarization period (during this week, participants were introduced to the study procedures, equipment, and exercises. They engaged in initial activities designed to help them understand the training regimen and become comfortable with the exercises they would be performing); Week 2 - Pre-test period; Weeks 3 to 10 - Training period; Week 11 - Post-test period. In addition, 3 months after post-test (follow-up) the participants were recruited to the school to reassess test results. Two weeks before the training period, a researcher communicated with the participants and standardized the training procedures. The weekly training sessions were co-created between the trainer and the participants, who were instructed to properly execute the prescribed exercises.

The study was registered and allocated by the Clinical Trials (IRCT20250316065103N1). Also, the study was approved by the University of Guilan (Iran) IR.GUILAN.REC.1403.169. All experiments were conducted in accordance with the relevant guidelines and regulations.

### Procedures

The participants underwent a total of 2 weeks of testing, which included both pre-tests and post-tests. To minimize the potential impact of circadian variations on the test results, the participants were tested at the exact same time of day (between 8 AM and 11 AM) and on the same day of the week as the pre-test session. The pre-test, post-test, and training programs were all conducted at the same school attended by the students. Additionally, the testing was conducted during the same training hour as the pre-test, ensuring consistency in the testing conditions at the school.

### Measurements

The participants’ height was measured using a wall-mounted stadiometer (Seca 222, Terre Haute, IN) and recorded to the nearest 0.5 cm. Their body mass was measured to the nearest 0.1 kg using a digital scale (Tanita, BC-418MA, Tokyo, Japan)^20^.

The following sections describe the testing procedures for assessing kyphosis, forward head posture, and shoulder alignment, which were accurately measured using a flexible ruler, goniometer, and “double square” tool. To enhance the reliability of these methods, all measurements were conducted by a highly skilled and competent researcher who is experienced in measuring posture. The researcher adhered to standardized protocols throughout the assessment process, ensuring consistency and accuracy across all measurements. This meticulous approach not only strengthens the validity of the findings but also reinforces the integrity of the study.

Kyphosis measurement: A flexible ruler was employed to measure the dorsal curvature of the spine due to its characteristics of being inexpensive, non-invasive, and quick. Participants were asked to uncover their upper bodies, allowing the researcher to observe and palpate the spine to identify the second and twelfth dorsal vertebrae. To locate the second dorsal vertebra, the participant was instructed to bend their head forward while standing. In this position, the researcher identified the most prominent vertebra, the seventh cervical vertebra, by touch. From there, two vertebrae below the seventh cervical vertebra were marked as the second dorsal vertebra. For the twelfth dorsal vertebra, the participant placed their hands on the edge of a table and shifted their weight forward into a semi-bent position. The researcher identified the twelfth vertebra by palpating the twelfth rib on both sides, following the rib’s path upward and inward until it disappeared into the soft tissue. If there was uncertainty regarding the location of the twelfth vertebra, the subject was asked to bend forward while the researcher placed their thumbs at the suspected location. The movement felt during bending and extension confirmed the exact position of the twelfth vertebra, as it is adjacent to the junction of the thoracic and lumbar vertebrae, with the first lumbar vertebra located just below it. Once the points were identified, they were marked with an easily erasable marker. All measurements were taken while the participants stood in a relaxed position, distributing their weight evenly between both feet and looking straight ahead. After marking the points, the flexible ruler was placed along the spine, conforming to its shape without any gaps. The marked points were then transferred to the ruler. The ruler was carefully removed, and the curvature was drawn on paper with a pencil, marking the identified points on the drawn curvature. The distance between the two points (L) and the deep of the curvature (H) were measured using the ruler. These measurements were inserted into the formula $\:{\uptheta\:}=4\text{A}\text{r}\text{c}\text{tan}2H/L$ to calculate the kyphosis angle^21^.

Forward head measurement; to measure the forward head angle using a goniometer, specific anatomical points were identified, including the tragus of the ear, the spinous process of the C7 vertebra, and the acromion prominence, which serve as reference points for assessing the position of the head and shoulders. The participant was placed in a natural standing position and instructed to focus their gaze on a point along the horizon. The goniometer was then positioned with its center at the tragus of the ear, one arm aligned parallel to an imaginary line drawn from the tragus to the C7 vertebra, and the other arm aligned with a horizontal line parallel to the ground. The angle obtained from this setup was recorded as the forward head angle. To minimize human error, each measurement was repeated three times, and the average value was calculated as the final data point. This method is widely used in corrective movement studies and postural analysis due to its simplicity, accuracy, and effectiveness in evaluating posture^22^.

Measuring the forward shoulder posture using the “double square” tool: the device utilized for measuring the forward shoulder position consists of a 40 cm composite ruler attached inverted to an additional surface, allowing for precise measurement of the distance from the anterior tip of the acromion to the wall. Studies have demonstrated that this method exhibits very high interrater reliability (ICC = 0.99 and SEM = 0.1 mm), underscoring the tool’s accuracy and efficiency in assessing forward shoulder position^23^. The location of the anterior tip of the acromion on both the right and left shoulders of the participate was marked by touch and with a permanent marker. participates placed their heels against a wall and were instructed to maintain a “military stance” to ensure stability and prevent changes in body position. The double square tool was positioned so that one square was tangent to the wall. The second square was gently adjusted to contact the anterior tip of the acromion. The distance between the wall and the acromion tip was recorded to the nearest millimeter. This measurement was repeated three times for each shoulder, and the average was recorded for statistical analysis. After recording values in the military position, the participate returned to a natural and relaxed position, and the measurement process was repeated. To achieve the military position, participates were instructed to: Stand straight with shoulders pulled back and chest opened. Tuck the chin in slightly and keep the head straight. Place feet firmly on the floor while keeping the knees in a natural position. In this stance, participates were encouraged to maintain natural body balance without excessive muscle tension or unnatural bending. The military position serves as a standard reference for comparing the subject’s natural position and helps prevent unwanted changes in body posture during the test^24^.

daily habits measurement: to assess students’ daily functional activities, a researcher-developed questionnaire based on the study of Emami, Shamsi Majelan and Norasteh (26) was utilized. The reliability of the questionnaire was evaluated using the Cronbach’s alpha method, which yielded a reliability coefficient of 0.82, indicating a high level of internal consistency. Validity was confirmed by teachers and experts in corrective movements, ensuring that the content accurately reflects the intended measurements of daily activities. The questionnaire comprised 19 questions organized into three dimensions, with each dimension containing a specific number of questions related to daily functioning. Additionally, several questions included visual aids to enhance understanding and engagement. Scores ranged from 0 to 19, with scores closer to 0 indicating poorer performance in daily activities, while scores closer to 19 reflected better performance. This structured approach provides valuable insights into the students’ daily lives, facilitating targeted interventions to improve their overall well-being^25^.

### Interventions

#### Postural education program

The training sessions incorporated in the current study are designed to address postural disorders, particularly among mothers and students, drawing from established “back school” programs prevalent in existing research. These health education initiatives aim to mitigate injuries and enhance the quality of life for individuals suffering from chronic musculoskeletal pain, notably spinal discomfort, by promoting appropriate activities of daily living. The program implemented in this study mirrors these objectives but is tailored to the audience: parents and guardians received educational materials over two sessions, while students were taught using simplified language for better comprehension^26^.

The content of the training is structured into several key modules. The first module, “Anatomy of the Human Body,” covers the structure and function of various body parts, including bones, joints, and the spine, supplemented by illustrative slides. The second module focuses on “Movement, Posture, Balance, and Body Awareness,” emphasizing concepts such as the center of gravity and its relation to posture and movement, as well as static and dynamic balance^26^.

The third module addresses “Sitting,” where participants analyze sitting positions in both classroom and home environments. This includes distinguishing between comfortable and correct sitting and exploring various sitting types, such as on the floor, at a desk, or in a chair. Practical exercises were conducted, where one student demonstrated different sitting positions while peers analyzed spinal alignment. Additionally, ergonomic solutions for computer desk setups were presented to mothers, encouraging students to optimize their workstations through simple adjustments, such as elevating the monitor or using a shoebox as a footrest^26^.

The fourth module, “Picking Up, Putting On, and Carrying a Backpack,” involved a hands-on analysis of commonly used items. Students practiced moving objects while the researcher monitored their techniques to prevent harmful movements. Discussions included the weight, size, and shape of backpacks, with individual assessments to adjust strap sizes and materials for optimal fit. Emphasis was placed on weight management, advising students on the organization of backpack contents—placing heavier items closer to the body—and demonstrating the correct lifting technique by recommending that backpacks be placed on elevated surfaces before being worn. To reinforce learning, students practiced various carrying styles, while peers provided feedback on their posture and technique^26^.

### Corrective games program

Table 2 provides a comprehensive overview of the protocol for a series of corrective games specifically designed to enhance physical fitness and promote postural alignment among participants. Each game targets distinct muscle groups crucial for maintaining proper posture, ensuring a well-rounded approach to physical development. The games are structured with clear objectives, allowing participants to understand the purpose of each activity. For instance, “Snake Crawling Forward - Tent on the Ball” focuses on stretching the large back, chest, and smaller muscles, while “Carrying a Book (Bag.) with the Head” emphasizes the stretching of the pectoralis major/minor and the internal rotators of the shoulder. By concentrating on these specific muscle groups, the games facilitate targeted improvements in posture and overall muscular balance. Each activity is designed for groups of participants, typically involving two groups of ten, fostering an environment of teamwork and cooperation. The playing areas are adequately sized (20 × 10 m) to accommodate the movements required in each game, ensuring safety and ample space for physical activity. The required equipment, such as Swiss balls, exercise bands, and volleyballs, is selected to enhance the effectiveness of each game while promoting engagement and enjoyment among participants. Moreover, the games include variations for progressive difficulty, enabling participants to gradually increase their challenge as they develop their skills and strength. For example, in the “Snake Crawling Forward” game, participants start with a 12-meter track length and progress to more complex variations involving obstacles and lunges over an eight-week period. This gradual increase in difficulty not only maintains participant interest but also ensures continuous improvement in their physical capabilities and postural awareness. Overall, this structured approach to corrective games not only fosters physical development but also enhances teamwork and encourages participants to be mindful of their posture during activities. By integrating these elements, the program ultimately contributes to improved musculoskeletal health, reinforcing the importance of posture in physical fitness and well-being^27^ (Table 2).

**Table 2:** Summarizes the protocol for a series of corrective games.

Game title	Objective	Number of participants	Playing area	Required equipment	Game description	Variations/progression
Snake Crawling Forward - Tent on the Ball	Stretching the large back, large chest, and small muscles	2 groups of 10	20 × 10	Swiss balls (size 45 or 55)	Participants are divided into two groups. They start 20 m apart, kneel, and place their forearms on the ball while maintaining a natural head and neck position. Subsequent participants pass over their partner to take position. The last member calls for movement, and the first group to cover the distance wins. The game is played in 2 rounds	Weeks 1–2: 12-meter track length; walking/running Weeks 3–4: 16-meter track with two 30-cm obstacles Weeks 5–6: 18-meter track with three 30-cm obstacles Weeks 7–8: 16-meter track with lunges maintaining correct posture
Carrying a Book (Bag…) with the Head	Stretching the pectoralis major/minor and internal rotators of the shoulder	2 groups of 10	20 × 10	One-meter-long stick, a book or object	Participants place a stick on their back and a book on their head. They move back and forth, passing the book to the next player. Each drop costs a point. The group that finishes faster with fewer errors wins. The game is played in 2 rounds	Weeks 1–2: 12-meter track length Weeks 3–4: 16-meter track with obstacles Weeks 5–6: 18-meter track with more obstacles Weeks 7–8: Emphasis on correct form
Passing the Ball Under the Bridge	Stretching the pectoralis major/minor, internal rotators of the shoulder	2 groups of 10	20 × 10	Volleyball or training ball (35 cm diameter)	Participants assume a crab position and pass the ball under their bodies to teammates. The last person moves to the front after catching the ball. The team that reaches the end first wins	Weeks 1–2: 12-meter path Weeks 3–4: 16-meter path with obstacles Weeks 5–6: 18-meter path with increased obstacles Weeks 7–8: Focus on maintaining form
Catapult - Middle	Strengthening the parallelogram muscles, middle/lower trapezius, external rotators	2–16	20 × 10	Spiked exercise ball (45 cm), exercise band (1–1.2 m)	One group holds an exercise ball with bands while the other group throws balls at them. Points are deducted for hits. The game is played in three sets, with each player throwing three times before switching groups. The group with the fewest hits wins	Weeks 1–4: Elbows flexed Weeks 5–8: Elbows opened for better hold on the ball
Cable Car - Crossing the River	Strengthening the parallelogram and trapezius muscles	1–16	20 × 10	Two ropes (15 m long)	Two ropes are fixed at a height. Participants hook an elastic to the rope and pull themselves along it, ensuring shoulders and head touch the ground before moving on. The first team to finish wins	Weeks 1–2: 6-meter path Weeks 3–4: 7-meter path Weeks 5–8: 8-meter path
Ant (Carrying Luggage)	Strengthening deep flexor muscles of the neck	2–16	20 × 10	Book or bag, exercise elastic (yellow and red)	Participants balance a book or bag on their heads while pulling an elastic band. They pass the item to teammates at the end of the path. The first team to finish wins, with penalties for drops	Weeks 1–2: Use yellow elastic, walk/run Weeks 3–4: Navigate over obstacles Weeks 5–6: More obstacles Weeks 7–8: Use red elastic with obstacles

### Statistical analysis

All values are presented as mean ± standard deviation (SD). The pre- and post-values for the dependent variables were analyzed to determine if the distributions were normal using the Shapiro-Wilk Normality test. Differences in all variables were analyzed using a 3 (group) x 3 (time) repeated measures ANOVA. When a significant F-value was achieved across time or groups, Bonferroni post-hoc procedures were performed to identify the specific pairwise differences. Additionally, the effects of training (effect size [ESs]) were calculated using Cohen’s d^28^. To address the issue of missing data, particularly in the context of potential participant dropout, multiple imputation techniques were applied to ensure that the analysis remained robust and valid. This method allowed for the estimation of missing values based on the observed data, thereby maintaining the integrity of the dataset and minimizing bias that could arise from incomplete data.

## Results

In the primary stage of the study, 68 male children recruited in the study and then based on inclusion and exclusion criteria 60 participants were included and allocated into 3 groups (*n* = 20 for each group). To be included in the final analyses, participants were required to complete all the training sessions and attend all assessment sessions. As a result of these requirements, no participants were excluded from the study. This indicates that all individuals who met the criteria were able to participate fully. Therefore, 20 participants for each group were included for final analysis (Flowchart).

**Figure:**
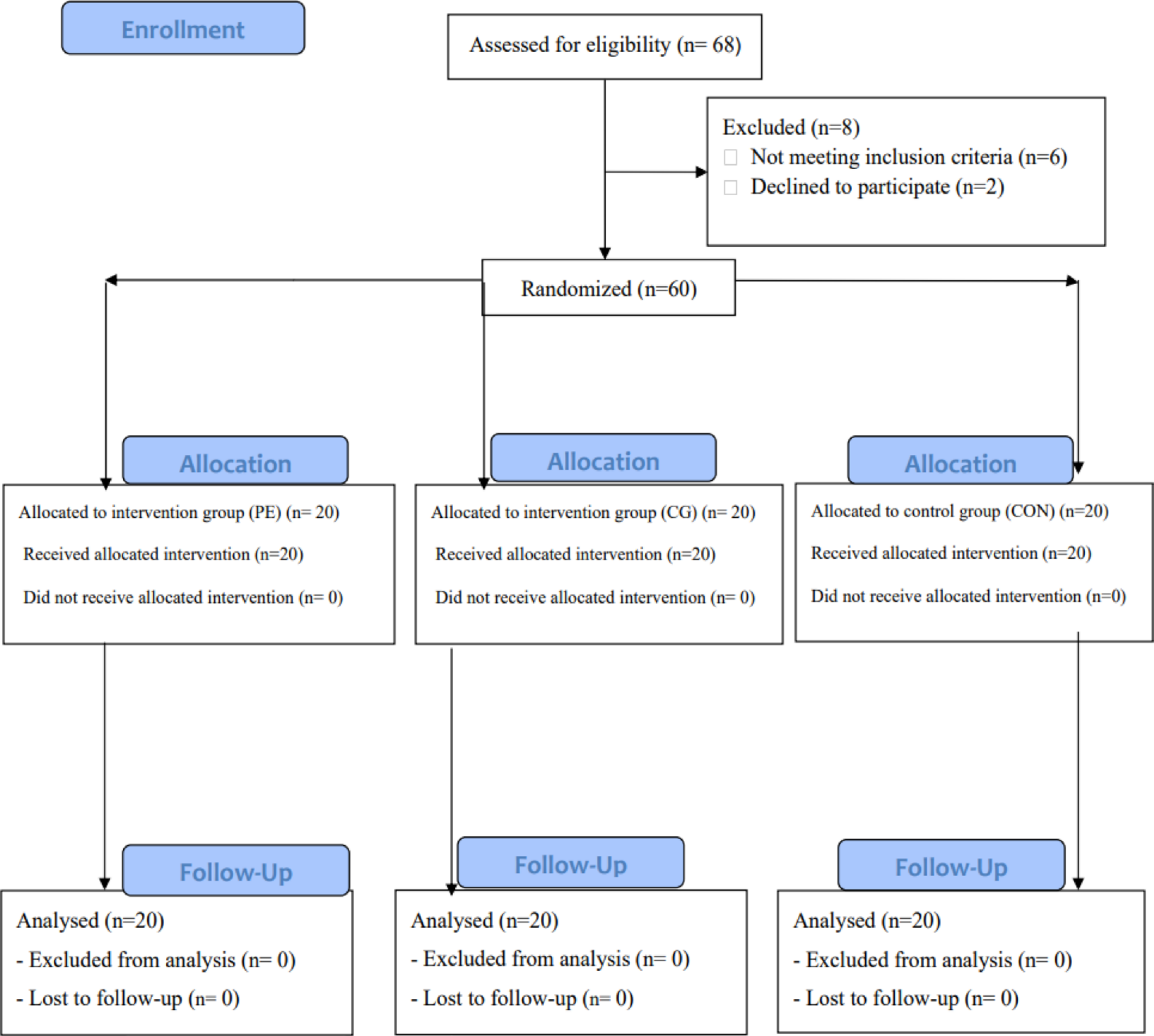
Flowchart of eligibility, inclusion and exclusion criteria, and analysis. CG: corrective games; PE: posture education.

### Flowchart around here

Before training, no significant differences were observed for the dependent variables kyphosis, forward head posture, forward shoulder posture, and daily habits between the groups (*p* > 0.05), indicating that they were comparable at baseline. No significant changes were observed in the CON for all variables after the training period (*p* > 0.05), as this group did not receive any intervention during that time. The test-retest reliability coefficient of these tests was *r* ≥ 0.89. To assess intraclass correlation coefficient (ICC), two measurements were made with 48 h apart and the ICC for the tests was *r* ≥ 0.89.

There was a significant interaction between group and time for kyphosis (F = 5.2, *p* = 0.01), forward head posture (F = 4.2, *p* = 0.02), forward shoulder posture (F = 3.9, *p* = 0.001), and daily habits (F = 6.1, *p* = 0.001).

Both the PE and CG showed significant improvements in kyphosis (*p* = 0.01 for PE, *p* = 0.02 for CG), forward head posture (*p* = 0.02 for PE, *p* = 0.04 for CG), forward shoulder posture (*p* = 0.001 for PE, *p* = 0.02 for CG), and daily habits (*p* = 0.02 for PE, *p* = 0.03 for CG) after an 8-week training intervention compared to the CON group. However, after a 3-month detraining period, the changes in both the PE and CG were found to be statistically insignificant. The p-values for kyphosis (PE = 0.23, CG = 0.33), forward head posture (PE = 0.56, CG = 0.36), forward shoulder posture (PE = 0.36, CG = 0.43), and daily habits (PE = 0.23, CG = 0.16) indicate that these differences were not significant (Table 3).

**Table 3:** Significant and effect size for the variables in the groups [*n* = 60].

Variables	Groups	Time point values (mean ± SD)			Statistic	Effect size (95% CI) and *p*-value			p-value
		Pre	Post	3-month post		Pre to post ES	p-value	Post to 3-month post ES	
Kyphosis	PE	35.14 ± 5.19	28.35 ± 6.14*	29.17 ± 5.34	G = 0.02	1.12 (0.37 to 1.86) d	0.001	− 0.14 (− 0.46 to − 0.23) a	0.34
	CG	34.79 ± 5.67	28.11 ± 5.76*	29.11 ± 6.11†	T = 0.01	1.21 (0.48 to 1.79) d	0.003	− 0.19 (− 0.56 to − 0.28) a	0.29
	CON	36.19 ± 5.37	35.76 ± 5.85	36.41 ± 5.11	G×T = 0.01	–		–	
Forward head	PE	36.44 ± 4.28	30.17 ± 5.47*	30.43 ± 5.47	G = 0.03	1.48 (0.36 to 1.86) d	0.002	− 0.21 (− 1.08 to − 0.68) b	0.49
	CG	35.86 ± 5.12	30.11 ± 5.6*	31.11 ± 4.61	T = 0.03	1.26 (0.26 to 1.56) d	0.001	− 0.24 (− 1.14 to − 0.62) b	0.26
	CON	34.98 ± 5.11	35.12 ± 4.97	35.16 ± 5.29	G×T = 0.02	–		–	
Forward shoulder	PE	16.43 ± 4.48	11.76 ± 4.12*	11.49 ± 4.46	G = 0.02	1.39 (0.14 to 1.54) d	0.001	− 0.23 (− 0.98 to − 0.77) a	0.46
	CG	16.11 ± 5.12	10.35 ± 4.43*	11.16 ± 5.18	T = 0.02	1.42 (0.16 to 1.63) d	0.002	− 0.43 (− 1.12 to − 0.64) b	0.36
	CON	15.98 ± 4.98	16.47 ± 4.36	16.49 ± 4.79	G×T = 0.001	–		–	
Pattern of daily activities	PE	9.11 ± 5.16	14.26 ± 4.19*	13.14 ± 5.72	G = 0.01	0.98 (0.38 to 1.4) d	0.001	− 0.24 (− 1.11 to 0.86) a	0.31
	CG	9.76 ± 5.19	15.06 ± 5.43*	14.43 ± 5.26	T = 0.001	1.01 (0.66 to 1.78) d	0.001	− 0.21 (− 1.1 to 0.89) a	0.28
	CON	10.11 ± 5.18	11.08 ± 4.98	10.87 ± 5.18	G×T = 0.001	–		–	

PE: Posture education; CG: Corrective games., CON; control; G: group; T: time; CI: confidence interval; a: trivial; b: small; c: moderate; d: large ES. *Significant differences compared to pre and CON (*p* < 0.05), † significant differences compared to post (*p* < 0.05).

## Discussion

This study was designed to examine the effect and durability of postural education and corrective games on the alignment of the thoracic and cervical spine and the daily habits in children. The findings of this study indicated that both PE and CG groups decreased kyphosis angle, forward head posture, forward shoulder posture and improved daily habits of children. Also, results indicated that following the 3-month follow period no significant changes were observed in the measured variables. These findings are in line with results of previous studies^29^ which examined the effects of PE or CG on spine posture and found significant effects.

In explaining the impact of the PE on children’s spines, it can be stated that this initiative functions as a targeted educational intervention designed to enhance awareness of proper body mechanics and postural alignment^12^. This educational framework focuses on increasing awareness regarding optimal postural alignment, rectifying postural deviations, and promoting sustainable movement patterns^15^. By fostering awareness of correct posture, the program aims to mitigate the risk of developing postural deformities such as kyphosis, forward shoulder posture, and forward head posture^12^. It emphasizes the importance of educating students about appropriate postural behaviors and the biomechanical principles governing body use in various environments^30^. Instruction encompasses a wide range of activities, from proper sitting techniques to ergonomic practices for carrying loads and utilizing technological devices. Integrated into physical education curricula, the PE utilizes sports and physical activities to reinforce correct movement habits^31^. Through this comprehensive approach, the program effectively equips children with the knowledge and skills necessary to maintain proper posture, thereby reducing the likelihood of spinal deformities and promoting overall musculoskeletal health.

Additionally, in discussing the effect of the CG on children’s spines, it can be noted that the training regimen for the corrective games group comprised a variety of movements, including activities such as “wooden houses,” “trying to get up,” “airplanes,” and “mice and cats,” all designed to elongate shortened muscles^16^. Additionally, exercises such as “carrying a tennis ball,” “judging a blackboard,” “pull-pull,” “broken knee,” and “trying to get up” were employed to enhance muscle strength^27^. These movements significantly contributed to the improvement of spinal posture complications^16^. A key mechanism behind the effectiveness of corrective games lies in their engaging nature and the collaborative group dynamics, which serve to motivate participants to engage in the exercises^32^. Corrective games represent a more accessible and effective approach for enhancing spinal posture. Their inherent motivational aspects, stemming from their enjoyable nature, make them particularly suitable for children, aiding in the correction of maladaptive spinal postures^17^.

Regarding the long-term sustainability of both programs, it can be stated that the focus is on correcting body alignment, strengthening the supporting muscles, and enhancing shoulder stability, while also emphasizing the importance of proper movement patterns^33^. These elements are crucial as they contribute to an increased awareness of body posture in a corrected and controlled manner, facilitated by the nervous system^34^. By fostering this awareness, the programs promote lasting effects on spinal health and overall musculoskeletal function^35^. The integration of these principles ensures that participants not only achieve immediate improvements but also develop the necessary skills and habits to maintain these benefits over time, ultimately supporting sustained spinal alignment and stability^33^.

Furthermore, the results of the study indicated that both the PE and CG exhibited improvements in the daily habits, with no notable difference in the effectiveness of each program in enhancing daily habits. These findings align with previous studies^36^, which investigated the effects of PE and CG on daily activity patterns and reported significant positive outcomes. This consistency in results suggests that both programs are effective in promoting healthier daily behaviors.

The PE plays a crucial role in shaping children’s behaviors in their daily lives, particularly through the enhancement of body awareness and the correction of posture. By integrating educational content focused on proper body mechanics, PE equips children with the knowledge and skills necessary to apply these techniques in various everyday activities, such as sitting, lifting, and using technology. One of the primary goals of PE is to enhance body awareness among children. This involves teaching them to recognize their body’s position in space and how it moves during different activities. For instance, when children learn about the importance of maintaining a neutral spine while sitting at a desk or lifting objects correctly, they begin to develop a conscious awareness of their posture. This awareness is critical because it empowers them to make informed choices about their body mechanics throughout the day. The effective application of body mechanics, as demonstrated in PE and CG classes, significantly improves the adjustments of the musculoskeletal system. By focusing on balance and the distribution of effort during daily activities, children can alleviate discomfort and potentially slow down degenerative processes in their bodies^37^. For example, when children are taught how to lift heavy objects using their legs instead of their backs, they not only reduce the risk of injury but also reinforce healthier movement patterns that can last a lifetime. Changing ingrained postural habits is inherently challenging, as these habits are often deeply rooted in both movement patterns and mental frameworks^38^. Educational interventions must create opportunities for children to reflect on their current postures and movements. This reflection is essential for fostering a deeper understanding of how their body mechanics impact their health and well-being.

Moreover, psychological and cultural factors play a significant role in shaping postural habits^37^. For instance, children may adopt certain postures based on what they observe in their peers or family members, which highlights the need for interventions to address these influences. By incorporating discussions about the importance of posture and the consequences of poor body mechanics into the curriculum, educators can help children recognize and challenge these cultural norms. For a habit to change, children must learn to observe and interpret the sensations produced by their movements. This is a fundamental goal of PE and CG interventions^39^. By engaging in activities that promote mindfulness and body awareness, children can become more attuned to how their bodies feel during different postural positions. This heightened awareness encourages them to adjust their behaviors in real-time, leading to healthier habits. The PE methodology encourages greater autonomy for children in addressing posture-related issues in their daily lives^37^. By empowering them with knowledge and practical skills, children can take ownership of their posture and movement patterns. This autonomy is crucial; when children feel capable of making positive changes, they are more likely to incorporate these lessons into their routines. The educational activities highlighted in this study are specifically designed to promote proper postures through habit modification, serving as a vital approach to health promotion^39^. These programs should be tailored to address the specific lifestyles and behaviors that contribute to or exacerbate health problems. For instance, incorporating lessons on ergonomics for computer use can directly impact how children interact with technology, promoting healthier habits that can reduce the risk of musculoskeletal issues.

Another significant aspect addressed in this study is the examination of the impact of time on the retention of knowledge and habits acquired following the intervention. Consistent with previous research^40,41^, our findings demonstrate that improvements in knowledge and habits were maintained compared to pre-test levels. Although our follow-up assessment occurred three months post-intervention, studies with extended follow-up durations—ranging from one year^42,43^ to eight years^44^—have also reported comparable results. This sustained improvement over time suggests a positive long-term effect of interventions on both knowledge and habits.

### Limitations and future scope

The study presents several methodological limitations that warrant careful consideration. Firstly, the findings are specific to male children aged 9–12, which raises important questions about the applicability of the results to other demographics. This narrow focus limits the ability to generalize the findings to female children or broader age groups, as different developmental stages may influence how children engage with and benefit from exercise programs. For instance, girls may exhibit different movement patterns, social influences, and motivations that could affect their postural habits and responses to educational interventions. Consequently, future research should aim to include diverse populations to assess whether similar outcomes can be observed across genders and age groups, thereby enhancing the external validity of the findings. Additionally, while the use of a questionnaire to assess daily activities is practical for systematically capturing subjective perceptions due to its ease of use and low cost this method introduces potential biases that could affect the study’s conclusions. The reliance on self-reported data means that responses are significantly influenced by the participants’ perceptions, cognitive abilities, and willingness to accurately reflect their behaviors, which can lead to over- or under-reporting of certain activities. Moreover, questionnaires are inherently limited in their ability to verify how well individuals translate theoretical knowledge into practical application. For example, while children may report understanding proper body mechanics, the questionnaire cannot assess whether they effectively incorporate this knowledge into their daily movements. A more robust evaluation could be achieved through methods such as video analysis of dynamic posture, providing objective data on how children implement learned techniques in real-world scenarios. This approach would not only enhance the accuracy of the findings but also offer deeper insights into the relationship between educational interventions and actual behavioral changes.

Future research should focus on specific interventions to enhance postural awareness and body mechanics among children. This includes developing gender-specific programs to address the unique needs of male and female children, as well as age-appropriate interventions for different developmental stages. Multimodal approaches that combine physical exercises with educational workshops on ergonomics could improve practical application. Incorporating technology, such as mobile apps or wearables, can provide real-time feedback to encourage better posture. Additionally, involving parents in these interventions may reinforce healthy habits at home. Longitudinal studies are needed to assess the long-term effectiveness of these interventions, while exploring cultural and environmental factors will enhance their relevance. By targeting these areas, future research can effectively promote healthy postural habits and prevent musculoskeletal issues in diverse populations.

## Conclusion

In conclusion, both the PE and CG interventions significantly reduced kyphosis angle, forward head posture, and forward shoulder posture, while enhancing daily activity patterns without notable differences in effectiveness. These findings highlight the importance of promoting awareness of proper body mechanics to prevent postural deformities. The sustainability of these improvements suggests that participants developed lasting skills for spinal health. Overall, the study underscores the value of integrating educational and engaging physical activities into curricula to support children’s musculoskeletal well-being.

## Data Availability

The datasets used and/or analyzed during the current study are available from the corresponding author on reasonable request.

## Abbreviations

PE: Posture education
CG: Corrective games
